# An algorithm to enumerate all possible protein conformations verifying a set of distance constraints

**DOI:** 10.1186/s12859-015-0451-1

**Published:** 2015-01-28

**Authors:** Andrea Cassioli, Benjamin Bardiaux, Guillaume Bouvier, Antonio Mucherino, Rafael Alves, Leo Liberti, Michael Nilges, Carlile Lavor, Thérèse E Malliavin

**Affiliations:** 1 Institut Pasteur, Structural Bioinformatics Unit, 25, rue du Dr Roux, Paris, 75015 France; 2 CNRS UMR3528, 25, rue du Dr Roux, Paris, 75015 France; 3 University of Campinas (IMECC-UNICAMP), Campinas-SP, 13083-859 Brasil; 4 LIX, Ecole Polytechnique, Palaiseau, 91128 France; 5 IBM TJ Watson Research Center, NY Yorktown Heights, 10598 USA; 6 Université de Rennes-I, Rennes, France

**Keywords:** Distance geometry, Branch-and-prune algorithm, Molecular conformation, Protein structure, Nuclear magnetic resonance

## Abstract

**Background:**

The determination of protein structures satisfying distance constraints is an important problem in structural biology. Whereas the most common method currently employed is simulated annealing, there have been other methods previously proposed in the literature. Most of them, however, are designed to find one solution only.

**Results:**

In order to explore exhaustively the feasible conformational space, we propose here an interval Branch-and-Prune algorithm (*i*BP) to solve the Distance Geometry Problem (DGP) associated to protein structure determination. This algorithm is based on a discretization of the problem obtained by recursively constructing a search space having the structure of a tree, and by verifying whether the generated atomic positions are feasible or not by making use of pruning devices. The pruning devices used here are directly related to features of protein conformations.

**Conclusions:**

We described the new algorithm *i*BP to generate protein conformations satisfying distance constraints, that would potentially allows a systematic exploration of the conformational space. The algorithm *i*BP has been applied on three *α*-helical peptides.

## Background

Protein structure determination is crucial for understanding protein function, as it paves the way to the discovery of new chemical compounds and of new approaches to control the biological processes.

The problem of protein structure determination is certainly a problem with multiple solutions, as proteins are flexible polymers. As most of the experimental techniques of the structural biology obtain measurements averaged on an ensemble of protein conformations, the usual approaches for structure determination intend to find an average structure or a set of conformations describing fluctuations around an average structure. A path intending to get a complete coverage of the conformational space, given a series of constraints, is usually not taken, although such an approach could provide precious information about the conformational equilibrium, which is essential in the function of many proteins, as the HIV protease [1].

An important class of experimental methods for protein structure determination is based on the measurement of inter-atomic distances and angles, such as Nuclear Magnetic Resonance (NMR) spectroscopy [2] and cross-linking coupled to mass spectrometry [3]. In NMR, distance intervals between hydrogens are determined from the measurement of nuclear Overhauser effects (NOE). The experimentally measured distances are then used as constraints for protein structure calculations. Pure *in silico* approaches have been also developed based on the use of inter-atomic distance constraints, such as homology modeling [4] or prediction of protein-protein complexes [5] and ligand poses [6].

The Distance Geometry Problem (DGP) [7,8] consists in identifying the sets of points which satisfy a set of constraints based on relative distances between some pairs of such points. The present work describes an algorithm developed to solve DGP in the context of protein structure determination: the points represent the protein atoms.

The DGP is a constraint satisfaction problem. Several approaches solve this problem by reformulating it [8] as a global optimization problem having a continuous search domain, and whose objective function is generally a penalty function designed to measure the violation of the distance constraints. Over the years, the solution of DGPs arising in structural biology have been typically attempted by Simulated Annealing (SA) approaches based on molecular dynamics [9]. Other proposed approaches are based on various optimization methods as in [10]. As all meta-heuristic approaches, SA may provide approximate solutions but does not deliver optimality certificates. In the case of protein structure determination, since the optimization problem is a reformulation of a constraint satisfaction problem, solutions given by SA can be successively verified by checking the violations of the distance constraints. However, additional solutions may exist but go undetected by SA. Thus, an algorithm for the systematic enumeration of the possible conformations of a given protein could find a widespread field of application. Branch-and-prune algorithms and similar were proposed in the general context of protein structure determination [11-16], (see also [8] and references therein). However, these studies primarily addressed the question of defining relative orientations of protein monomers in symmetric oligomers, not the determination of all possible conformation of a polypeptide chain with a very large number of degrees of freedom from distance constraints. Systematic exploration was proved to be useful in the case of residual dipolar couplings (RDC) constraints [17], for exploring the sidechains conformations [18,19] and for assignment of NOEs, provided that the structure is known [20]. For the structure determination from RDCs, it has been shown [21] that when using RDCs but only sparse NOEs the problem can be solved in polynomial time. Such approaches have also been used for structure determination in X-ray crystallography for non-crystallographic symmetry by orienting and translating symmetric protein subunits [22]. To the best of our knowledge, in this paper we present the first application of a Branch-and-Prune algorithm to the problem of full protein structure determination based on unambiguous distance information.

**Figure 1:**
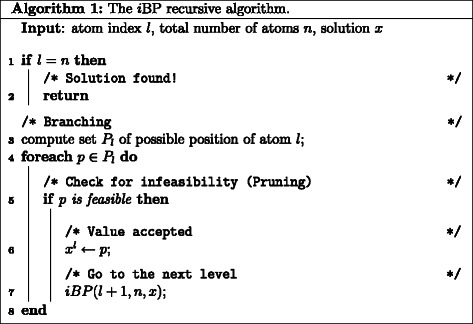
**The** ***i*** **BP recursive algorithm.** Description of the *i*BP algorithm.

**Figure 2:**
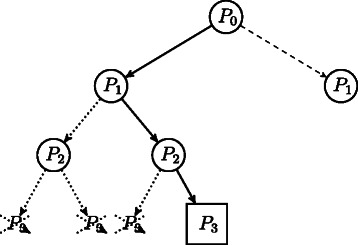
**The branch-and-prune search tree.** Example of branch-and-prune search tree exploration. With solid line, we depict the path currently in use, with dotted arcs pruned paths, and with dashed arcs paths not yet explored. The squared node corresponds to a feasible solution.

Under certain conditions, DGPs can be discretized [23] (see below), which means that the search domain for the corresponding optimization problem can be reduced to a discrete set, which has the structure of a tree. The discretization makes the enumeration of the entire solution set of DGP instances possible. This is important when the experimental constraints do not specify the protein conformation uniquely, i.e., more than one conformation satisfies all constraints. For solving discretized DGP, we employ an *interval* branch-and-prune (*i*BP) algorithm [24], which is based on the idea of recursively exploring the tree while generating new candidate atomic positions (branching phase) and to verify the feasibility of such positions (pruning phase) (Figure 1). By making use of pruning devices, branches rooted at infeasible positions can be discarded from the tree, so that the search can be reduced to the feasible parts of the tree (Figure 2). Pruning devices can be conceived and integrated in *i*BP to improve the performances of the pruning phase and thus of the algorithm.

In the present work, we first describe the branching phase and the pruning devices used to determine the solutions to the Distance Geometry problem. Then, an overall view of the method is given along with the use of the branching and pruning devices at different steps and the complexity of the algorithm is analyzed. We finally illustrate the algorithm application with three proteins for which *α*-helical regions are known along with few long-range NMR constraints (ie. constraints measured between residues *i* and *j* such that |*i*−*j*|>3 in the protein sequence). The obtained conformations display good stereochemical quality parameters, and the conformational space explored is larger than the one sampled with traditional optimization methods such as simulated annealing.

## Methods

In order to sample the conformational space of a protein, we use a Branch-and-Prune algorithm to build a tree in which each node represents a solution for one atomic position. We limit ourselves in the present work to the calculation of the backbone and C *β* atomic coordinates.

The constraints used to generate atomic coordinates along the Branch-and-Prune algorithm are the following:

1. covalent distance constraints corresponding to bond lengths and bond angles, whose values are derived from high-resolution small molecule X-ray crystal structures [25];
2. NMR distance constraints;
3. van der Waals radii of atoms between non-bonded atom pairs (*i*,*j*): a fraction of the sum of the van der Waals radii of each atom provides a lower bound to the corresponding inter-atomic distances:
  (1) $$ d_{ij}\geq \sigma (r^{vdw}_{i} + r^{vdw}_{j}),  $$
  where *σ*∈ [ 0,1], and is typically around 0.85. The values for the radii are given in Table 1 [26,27]. These lower bounds apply only in the cases where no larger lower bound has been determined from NMR distance constraints;

**Table 1:** **Van der Waals radii (see [** 26 **] and [** 27 **])**

**atom**	**O**	**H**	**C**	**N**
*r* ^*vdw*^ (Å)	1.4	1.0	1.7	1.5
4. distances derived from the backbone torsion angles *ϕ* and *ψ*;
5. hydrogen bonds in *α*-helix;
6. amino-acid chirality;
7. *α*-helix geometry.

**Figure 3:**
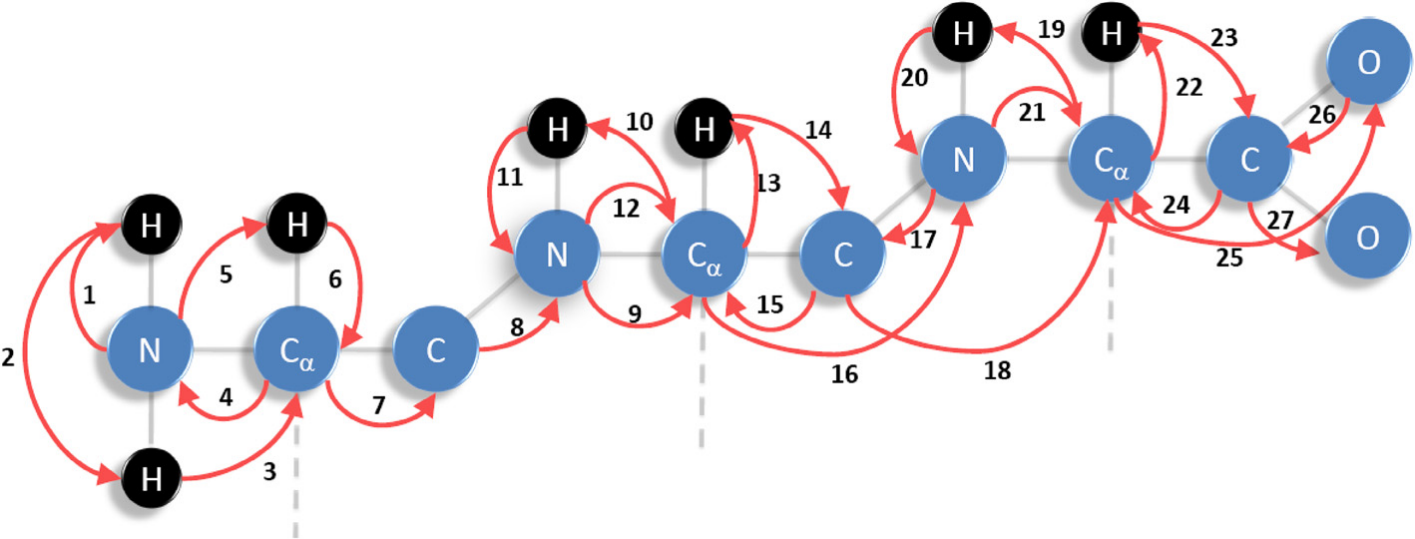
**Order** ***P***_***ato***_ **of the atoms parsed during the branch-and-prune algorithm.**

The atom coordinates are calculated, one by one, following the atom order *P*_ato_ described in Figure 3 and previously proposed in [24]. In this order, some atoms are repeated to insure that any entered atom is defined by distance constraints with respect to three preceding atoms in *P*_ato_ [24]. The carbonyl oxygens and the atoms C *β*, which were not present in the order *P*_ato_, are calculated separately.

Then, the tree is built using a recursive procedure to create each node of the tree. This procedure is called branching phase. The created nodes are then submitted to the pruning devices in order to decide whether the node should be kept or removed. If the node is removed, the possible branches starting from this node are also pruned. A pruning device is responsible for checking whether a partial solution is feasible, i.e. to check whether a set of embedded atoms fulfill the constraints (1)-(7) described above.

In the following, we describe the branching phase and the pruning devices. Then, the complexity of the algorithm is described from a theoretical point of view, before presenting some application cases.

### Branching devices

The tree parsed during *i*BP is formed by nodes, each corresponding to one set of atomic coordinates from the order *P*_ato_ (Figure 3) [24]. At each level of the tree, the atomic coordinates of the corresponding atom are calculated by making use of a recursive procedure, called branching phase. The current atom position is defined by distance constraints to three other atoms. These distances are obtained from the constraints (1-3) described above: (1) the covalent constraints, (2) the NMR distance constraints, (3) the van der Waals radii.

**Figure 4:**
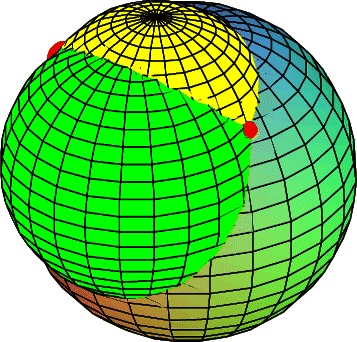
**Intersection of three spheres.** Intersection of three spheres, colored in yellow, green and cyan. The two points produced by the intersection are indicated with red spots.

If the distance constraints specify a unique value rather than an interval, this signifies that the distances to three immediate predecessors from the current vertex are known: these are the centers of the three spheres, and the distances are the radii of these spheres. The position of the current vertex/atom is thus defined by the intersection of three spheres, so there are at most two solutions for the current atom position: this is called a 2-branching situation (Figure 4).

**Figure 5:**
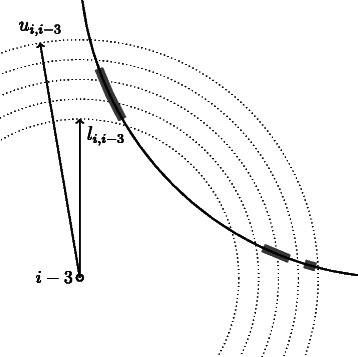
**Discretization of the distance constraints.** An example of discretization of the distance *d*_*i*,*i*−3_. The solid circle represents the result of the intersection of the spheres centered in *i*−1,*i*−2 with radii *d*_*i*,*i*−1_,*d*_*i*,*i*−2_, respectively. The distance *d*_*i*,*i*−3_ is discretized accordingly to Equation 2 with *b*=5: dotted circles represent the intersections of spheres centered in *i*−3 with radii in $\tilde d_{i}$ with the plane containing the *i*−3,*i*−2 and *i*−1. Thick gray arcs represent the feasible regions for the atom *i*.

When a distance is not uniquely defined, but rather defined by lower and upper bounds, i.e. *d*_*i*,*j*_∈[*l*_*i*,*j*_,*u*_*i*,*j*_], this distance is uniformly discretized by sampling *b*≥1 values in [*l*_*i*,*j*_,*u*_*i*,*j*_], as depicted in Figure 5.

(2) $$ \tilde d_{i}=\left\{ l_{i,i-3} + (t-1)\frac{(u_{i,i-3}-l_{i,i-3})}{b} : t=1,\ldots,b\right\}.  $$

In this case, we have a b-branching situation.

The algorithm used for calculating the atom coordinates is then applied to each set of $\tilde {d}_{i}$ values sampled for the distance constraints. The choice of the *discretization factor* *b* is a crucial point: a small value might lead to an infeasible problem because we may not select any feasible distance; a larger value increases the computational burden. In general, the finer the discretization, the more accurate the computation is, but it is not trivial to figure out the optimal value for *b*. One way to choose *b* is to consider that the number of nodes in the search tree is bounded by 3+(2^*l*^ *b* ^*k*^), where *l* is the number of tree levels where we have a 2-branching situation, and *k* is the number of tree levels where we have a b-branching situation [28]. Appropriate values of *b* should result in a manageable number of nodes.

Given the position of the three previous atoms *k*−3, *k*−2, *k*−1 in the order *P*_ato_ and given the constraints to these atoms of the atom *k* to be embedded, the position of *k* is calculated by a recursive matrix multiplication by making use of the set of distances *d*={*d*_*k*,*k*−1_,*d*_*k*,*k*−2_,*d*_*k*,*k*−3_} between the previous atoms and *k*. Although there are several methods to compute sphere intersections [29], in our experience, the best trade-off between efficiency and numerical stability is given by the use of recursion matrices [23], and of the two following angles: (i) the torsion angle *ω*_3_ formed by atoms {*k*,*k*−1,*k*−2,*k*−3} which depends on the distance between *k* and *k*−3, (ii) the angle *θ*_2_ formed by atoms {*k*,*k*−1,*k*−2}.

The recursion is applied through the equation:

(3) $$ \begin{aligned} \left[\begin{array}{c} x_{k} \\ y_{k} \\ z_{k} \\ 1 \end{array} \right] &= B_{1} B_{2} B_{3} \ldots B_{k}(d,\sigma) \left[\begin{array}{c} 0 \\ 0 \\ 0 \\ 1 \end{array}\right]\\ &= Q_{k-1}B_{k}(d,\sigma) \left[\begin{array}{c} 0 \\ 0 \\ 0 \\ 1 \end{array}\right] = Q_{k} \left[\begin{array}{c} 0 \\ 0 \\ 0 \\ 1 \end{array}\right], \end{aligned}  $$

where:

(4) $$ {\fontsize{7}{6}\begin{aligned} B_{k} (d,\sigma) = \left[\begin{array}{cccc} -\cos\theta_{2} & -\sigma \sin\theta_{2} &0& -d_{k,k-1}\cos\theta_{2}\\ \sigma\sin\theta_{2}\cos\omega_{3} & -\cos\theta_{2}\cos\omega_{3} & -\sin\omega_{3}& \sigma d_{k,k-1} \sin\theta_{2}\cos\omega_{3}\\ \sigma\sin\theta_{2}\sin\omega_{3} & -\cos\theta_{2} \sin\omega_{3}&\cos\omega_{3} &\sigma d_{k,k-1} \sin\theta_{2}\sin\omega_{3}\\ 0 & 0& 0&1 \end{array}\right], \end{aligned}}  $$

and *σ*∈{+1,−1}. The series of recursion matrices is initialized as:

(5) $$ \begin{aligned} B_{1}= \left[\begin{array}{cccc} 1 & 0& 0&0\\ 0 & 1& 0&0\\ 0 & 0& 1&0\\ 0 & 0& 0&1 \end{array}\right], B_{2}= \left[\begin{array}{cccc} -1 & 0& 0& -d_{2,1}\\ 0 & 1& 0&0\\ 0 & 0& -1&0\\ 0 & 0& 0&1 \end{array}\right],\\ B_{3}= \left[\begin{array}{cccc} -\cos\theta_{3} & -\sin\theta_{3} &0& -d_{3,2}\cos\theta_{3}\\ \sin\theta_{3}& -\cos\theta_{3} & 0 & d_{3,2} \cos\theta_{3}\\ 0 & 0& 1&0\\ 0 & 0& 0&1 \end{array}\right]. \end{aligned}  $$

*d*_2,1_ being the distance between the first and the second atom, and *d*_3,2_ the distance between the third and the second atom in the order *P*_ato_.

The total number of *B*_*k*_ matrices to be calculated along the parsing of the tree is bounded by 2∣ *P*_ato_ ∣*b*, where ∣ *P*_ato_ ∣ is the size of the ordered atom list *P*_ato_. The product *Q*_*k*−1_ *B*_*k*_ is calculated in two steps: (1) the fourth column of *Q*_*k*_, which gives us the coordinates of *k*, is computed; (2) only if *k* is not pruned, the three remaining columns are computed.

We must distinguish two cases when embedding an atom *k*. If it is the first appearance of *k* in *P*_ato_, we use equation 3 to compute all possible embeddings of *k* for *σ*∈{+1,−1} and the set of distances *d*. If it is not the first appearance of *k* in *P*_ato_, we need to take into account the fact that numerical instabilities generate matrices which will lead to slightly different coordinates for *k* than those computed the first time. In order to decrease the impact of these numerical errors, we compute the set of distances *d*, the angles *θ*_2_,*ω*_3_ and for *σ*∈{+1,−1} the corresponding matrices *B*_*k*_(*d*,+1),*B*_*k*_(*d*,−1), which lead to two possible embeddings of *k* (Equation 3), as *k* ^+^=*Q*_*k*−1_ *B*_*k*_(*d*,+1) and *k* ^−^=*Q*_*k*−1_ *B*_*k*_(*d*,−1). We choose the value of *k* that yields the updated coordinates of *k* being the closest to the previous coordinates of this atom.

Each carbonyl oxygen O ^*i*−1^ is uniquely determined for residue *i*, once *C* ^*i*−1^, *N* ^*i*^ and *H* ^*i*^ have been embedded, since these atoms are all part of the peptide plane [30]. As is common practice (see, e.g., [31-33]), we fix here the torsion angle *ω* of the peptide plane to -180° or 0°. In a previous implementation [34], the positions of the carboxylic oxygens were not stored. Although this approach leads to memory savings, the availability of carboxylic oxygen positions can improve the definition of the *α*-helix secondary structure.

The positions of the carbonyl oxygens are thus now calculated in the following way. If *k*=*O* ^*i*−1^ is the carboxylic oxygen atom located at the vertex *k*, and {*v*_1_,*v*_2_,*v*_3_} are the vertices corresponding to atoms {*C* ^*i*−1^,*N* ^*i*^,*H* ^*i*^}, belonging on the same peptide plane *π*, we denote *n*_*π*_ the normal vector to *π*. The coordinates of *k* can then be computed by solving the following non-linear system:

(6) $$ \left\{ \begin{array}{ll} \| k - v_{i} \|^{2} = d_{ki}^{2}, & i=1,2,3\\ n_{\pi}^{T} (v_{1} - k) = 0& \end{array} \right..  $$

where *d*_*ki*_ are the distances between atoms *k* and *i*. Using an approach similar to those employed in [35], we obtain the equivalent linear system:

(7) $$ \left\{ \begin{array}{l} 2 (v_{2} - v_{1})^{T} k = d_{k1}^{2} - d_{k2}^{2} -\|v_{1}\|^{2} +\|v_{2}\|^{2}\\ 2 (v_{3} - v_{1})^{T} k = d_{k1}^{2} - d_{k3}^{2} -\|v_{1}\|^{2} +\|v_{3}\|^{2}\\ n_{\pi}^{T} (v_{1} - k) = 0 \end{array} \right.  $$

The parameter *d*_*k*1_ is the length of the bond connecting *O* ^*i*−1^ and *C* ^*i*−1^, the parameters *d*_*k*2_ and *d*_*k*3_ are the distances between *k*=*O* ^*i*−1^ and *N* ^*i*^, *H* ^*i*^, calculated from bond angles and bond lengths between atoms of the peptide plane, and the angle *ω* of 180° in a *trans* peptide plane. The case of the *cis* peptide plane can be treated in the same way, modifying the value of *ω* to be 0°.

Following the idea proposed for carbonyl oxygens, the coordinates *k* of a *C* *β* atom can be computed from previously calculated atoms, because the four distances of *k* to atoms {*v*_1_=*C* *α*,*v*_2_=*H* *α*,*v*_3_=*N*,*v*_4_=*C*} are exactly known, and because these five atoms are not coplanar. The coordinates *k* are calculated by solving the linear system:

(8) $$ \left\{ \begin{aligned} 2 (v_{2} - v_{1})^{T} k = d_{k1}^{2} - d_{k2}^{2} -\|v_{1}\|^{2} +\|v_{2}\|^{2}\\ 2 (v_{3} - v_{1})^{T} k = d_{k1}^{2} - d_{k3}^{2} -\|v_{1}\|^{2} +\|v_{3}\|^{2}\\ 2 (v_{4} - v_{1})^{T} k = d_{k1}^{2} - d_{k4}^{2} -\|v_{1}\|^{2} +\|v_{4}\|^{2} \end{aligned} \right.  $$

The parameter *d*_*k*1_ is the length of the bond connecting *k*=*C* *β* and *C* *α*, the parameters *d*_*k*2_, *d*_*k*3_ and *d*_*k*4_ are the distances between *k*=*C* *β* and *H* *α*, *N*, *C*, calculated from bond angles and bond lengths between these atoms.

### Pruning devices

Once the set of possible coordinates of the atom *k* has been determined in the branching phase described above, pruning devices are used to check whether the coordinates of *k* are feasible. In some cases described below, the coordinates of *k* along with the coordinates of previously embedded atoms are checked together. If the check is negative, the solution obtained for *k* is discarded, which prunes all tree branches originating from the node *k*. In this section, we present the pruning devices used to accept or discard the coordinates of the atom *k* generated by the branching devices. The pruning device applies all these tests as soon as the involved atoms have been embedded.

#### Direct distance feasibility (DDF)

As the coordinates for an atom *k* are determined, we first check that all distances between *k* and the other embedded atoms respect the given lower and upper bounds arising from the constraints (1-3) listed in section “Solving the DGP with *i*BP”.

#### Torsion angle feasibility (TAF)

The values of the backbone torsion angles *ϕ*,*ψ*, are used as a pruning device, checking whether they are located in the permitted regions of the Ramachandran plot. The pruning device, first introduced in [34], is implemented in the following way. The torsion angle *ξ*_*ijkl*_ defined by a quadruple of atoms {*i*,*j*,*k*,*l*} falls into a domain *Ξ*_*ijkl*_, up to a certain tolerance *ε*_*t*_>0. In general, *Ξ*_*ijkl*_ is the union of *κ* dis-joined intervals, i.e.

(9) $$ \Xi_{ijkl} = \bigcup\limits_{c=1}^{\kappa} \Xi_{ijkl}^{c}  $$

From the bounds on a torsion angle *ξ*_*ijkl*_ it is possible to derive bounds on the distance *d*_*il*_, noticing that

(10) $$ d_{il}(\xi_{ijkl}) = \sqrt{d_{ij}^{2} + d_{lj}^{2} - 2(\cos(\xi_{ijkl})\sqrt{ef} + bc) d_{ij}d_{lj} },  $$

where:

(11) $$ \begin{aligned} b&= \frac{1}{2}\frac{d_{lj}^{2} + d_{jk}^{2} - d_{lk}^{2}}{d_{lj}d_{kj}} \\ c&= \frac{1}{2}\frac{d_{ij}^{2} + d_{jk}^{2} - d_{ik}^{2}}{d_{ij}d_{jk}}\\ e&= 1-b^{2}, f= 1-c^{2}.\\ \end{aligned}  $$

Taking the maximum and minimum values of *d*(*ξ*_*ijkl*_) for *ξ*_*ijkl*_∈*Ξ*_*ijkl*_, we obtain an interval [ *l*_*il*_,*u*_*il*_] for the distance *d*_*il*_. The sign of the angle *ξ*_*ijkl*_ is used as an additional pruning criterion along with the *d*_*il*_ interval.

#### Dijkstra shortest-path (DSP)

As introduced in [23], we can exploit the fact that the distances are Euclidean to improve the *i*BP pruning capabilities. We extend and generalize the procedure presented in [36] in the following way. We introduce an auxiliary graph *G* ^+^ with the same topology as the graph connecting the atoms in the protein, but such that the weight of each edge (*i*,*j*) is the upper bound of the distance *d*_*ij*_. For every pair of atoms *i*,*j*, the shortest-path between *i*,*j* in *G* ^+^ is a valid over-estimate of *d*_*ij*_. Thus we used an all-to-all shortest-path algorithm, the Floyd-Warshall algorithm [37], to refine the upper bound for each pair of atoms.

The Dijkstra Shortest-Path pruning device uses the refined upper bounds of inter-atomic distances in the following way. According to Lemma 4 in [23], for an atom *k* and for each atom pair *i*,*j* such that *i*<*j*<*k* in the order *P*_ato_ and for which *d*_*ik*_ is known, the embedding of *k* can be pruned if:

(12) $$ \|i-j\| - d_{ik}> u_{jk}  $$

where *u*_*jk*_ is the upper bound of the atom pair (*j*,*k*) obtained using the Floyd-Warshall algorithm [37].

#### Chirality (CHI)

The pruning of atom coordinates through the amino-acid chirality is implemented through the so-called CORN rule of thumb: in amino acids, the groups COOH, R (sidechain), NH2 and H are bonded to the chiral center C *α* carbon. Starting with the hydrogen atom away from the viewer, if these groups are arranged clockwise around the C *α* carbon, then the amino-acid is in the D-form. If these groups are arranged counter-clockwise, the amino-acid is in the L-form. The CORN rule was restated by imposing that the torsion angle defined by the atoms *C*,*C* *β*,*N*,*H* *α* of residue *i* for the D-form or *C*,*N*,*C* *β*,*H* *α* of residue *i* for the L-form, is positive.

#### *α*-helix secondary structure

We proposed the use of *α* helix information as a pruning device in the context of the *i*BP algorithm first in [34]. The *α* helix location can be determined from an analysis of the NMR chemical shifts by TALOS [38]. Four criteria are used to enforce the formation of an *α* helix: (i) the formation of backbone hydrogen bonds between amide hydrogens and carbonyl oxygens, (ii) the alignment of the amide and carbonyl functions checked by a qualitative condition on the energy of the hydrogen bond, (iii) the definition of backbone *ϕ* and *ψ* torsion angles already described in the Torsion Angle Feasibility, (iv) the definition of three additional angles *θ*, *θ*’ and *θ*” similar to the ones introduced by Grishaev et al. [39].

On a sequence of *m*+1 contiguous residues *I*_*α*_={*i*,*i*+1,…,*i*+*m*} forming an *α* helix, for any pair of residues (*i*−4,*i*) belonging to *I*_*α*_, the lower and upper bounds on the distance between the carboxylic oxygen *O* ^*i*−4^ and the amide hydrogen *H* ^*i*^ should be compatible with the formation of an hydrogen bond. The upper and lower bounds are defined in an input parameter file of *i*BP, and were set to 1.9 and 3.0 Å in the present work.

The condition checking the alignment of atoms involved in the hydrogen bond is implemented by calculating a local energy information defined in the DSSP package [40]:

(13) $$  q_{1}q_{2}\!\left[ \frac{1}{d_{O_{i-4}N_{i}}}\! +\! \frac{1}{d_{C_{i-4}H_{i}}}- \frac{1}{d_{O_{i-4}H_{i}}}\! - \!\frac{1}{d_{C_{i-4}N_{i}}} \right]\cdot f< -0.5,  $$

with *q*_1_=0.42,*q*_2_=0.2 and *f*=332, and *d*_*AB*_ correspond to the distance between atoms *A* and *B*.

The last criterion enforces the angles *θ*, *θ*’, *θ*” to be respectively into the interval values 0/70°, 0/90° and 110/180°.

### Implementation details

In this section we provide an overview of the main implementation features. The *i*BP algorithm has been coded in C++ with extensive use of template meta-programming [41], STL [42,43], and BOOST (www.boost.org). Linear systems, as for instance (7), are solved using the LAPACK library [44].

Discretizable DGP instances were represented by simple weighted undirected graphs *G*=(*V*,*E*,*d*), which were handled by the Boost Graph Library (BGL) [45]. The points in $\mathbb {R}^{3}$ were represented using the Boost Geometry Library (also known as Generic Geometry Library, GGL: www.boost.org).

Constraints on distances, angles or energy are typically expressed by enforcing a variable *x* to take values in a domain , which is generally the union of intervals and singletons:

(14) $$ \mathcal{D} =\left\{ \bigcup_{j=1}^{m} \bar x_{j} \right\}\cup \left\{ \bigcup_{i=1}^{k} \left[{x_{i}^{l}},{x_{i}^{u}}\right]\right\}.  $$

The Boost Interval Library (BIL – see [46,47]) was used to store such representation, and to perform basic operations for intervals and singletons. On top of the BIL, we define the type domain which contains a set of intervals and operations as intersection, scaling, etc. The BIL allows also to select the underlining data format for the interval (single/double precision real, integer).

## Theory

In this section we give some details about the worst-case asymptotic complexity behavior of the *i*BP algorithm. The description given above includes many details which are useful for finding the structure of proteins but which somewhat complicate the precise mathematical treatment. We first give a very brief abstract description of the *i*BP and of the formal problem it solves, and then proceed to discuss its complexity.

Formally speaking, the DGP is the following decision problem: given an integer *K*>0, a simple undirected graph *G*=(*V*,*E*) and an edge weight function $d:E\to \mathbb {R}_{+}$, is there a realization $x:V\to \mathbb {R}^{K}$ such that for each {*u*,*v*}∈*E* we have ∥*x*_*u*_−*x*_*v*_∥_2_=*d*_*uv*_? Note that we are writing *x*_*u*_ for *x*(*u*) and *d*_*uv*_ for *d*(*u*,*v*). We also remark that in the more “applied” interpretation given in the preceding section, the range of the edge function *d* is $\mathbb {IR}_{+}$, i.e. the set of all non-negative closed real intervals, and *K*=3. The DGP is **NP**-hard for any *K*>1 and **NP**-complete for *K*=1 [48]. Since we are interested in finding *all* solutions of the DGP rather than just one, we denote by *X* the set of all realizations of *G*.

### Assumptions on the DGP input data

In fact, due to the fact that our data come from a protein structure setting, we can also make the following assumptions about *G* and *d*:

1. there is an order 1,2,…,*n* on the vertices such that 1,2,3 is a triangle in the graph *G* and, for each vertex *v*>3, *v* is adjacent to *v*−1,*v*−2,*v*−3;
2. the set of edges *E* can be partitioned in two subsets *E*_*D*_ and *E*_*P*_, such that *E*_*P*_ consists of all edges {*u*,*v*} with *v*>4 and |*v*−*u*|>3, and $E_{D}=E\smallsetminus E_{P}$;
3. *E*_*D*_ can be further subdivided in $E_{D}^{\prime }$ and $E_{D}^{\prime \prime }$, so that $E_{D}^{\prime \prime }$ consists of all edges {*u*,*v*} with |*v*−*u*|=3, and $E_{D}^{\prime }=E_{D}\smallsetminus E_{D}^{\prime \prime }$;
4. the distance function *d* is such that: (a) *d*_*uv*_ is a scalar for each $\{u,v\}\in E^{\prime }_{D}$; (b) *d*_*uv*_ consists of a discrete set of *b* scalars for each $\{u,v\}\in E^{\prime \prime }_{D}$; (c) *d*_*uv*_ is a general interval for all {*u*,*v*}∈*E*_*P*_.

We remark that the above definitions can be appropriately extended to Euclidean spaces of any dimension *K*>0, not just *K*=3. We call *E*_*D*_ the *discretization edges* and *E*_*P*_ the *pruning edges*. Discretization edges ensure that the graph *G* is rigid, which implies that there are finitely many realizations of *G* in $\mathbb {R}^{K}$. Pruning edges make some of those realizations infeasible, and thereby make the solution set *X* smaller. A few remarks are in order:

- we consider that distances which are known because of covalent bond relations are sufficiently precise to be represented by a scalar;
- we consider that distances which are known from NOESY (or other) experiments can be represented by intervals;
- we assume that a limited number of the intervals can be discretized into sets containing a finite number *b* of values within the intervals;
- the edges in $E_{D}^{\prime }$ represent atom pairs of the form {*v*,*v*−1} or {*v*,*v*−2} for any *v*>2: these are involved in covalent bonds;
- the edges in $E_{D}^{\prime \prime }$ represent atom pairs which are assigned a certain number *b* of possible values (optionally *b*=1 for certain pairs);
- the edges in *E*_*P*_ represent atom pairs for which the distance might be a general interval.

We remark that the order on *V* was initially intended to follow the protein backbone [49], but new orders which better exploit the hydrogen atoms in or close to the backbone have been defined in [50,51]: these are the orders on which the above assumptions are based.

The DGP with the restrictions above, but where all intervals are replaced by scalars, is called DISCRETIZABLE MOLECULAR DGP (DMDGP). Both the DMDGP and its generalization to any *K* (denoted by ^K^DMDGP) are **NP**-hard [52,53]. The problem defined above, involving intervals, obviously contains the DMDGP as a sub-case and is hence also **NP**-hard by inclusion.

### When all distances are precise

We first focus on the simplest case, where all intervals are replaced by scalar values. Then $d:E\to \mathbb {R}_{+}$, and *b*=1. In this simplified setting, the *i*BP is simply called BP [52], and the order on *V* is called a *contiguous trilateration order* [54] or a *DMDGP order* [55].

The BP can be defined as a recursive procedure: assuming we already found a realization *x*_1_,…,*x*_*v*−1_ for the vertices 1,…,*v*−1, and that we mean to find a consistent realization *x*_*v*_ for *v*, the discretization edges *E*_*D*_ guarantee that there will be at most two positions for *x*_*v*_ compatible with the distances restricted to *E*_*D*_ [49]. This can be intuitively understood in $\mathbb {R}^{3}$ by considering the intersection of three spheres centered at *x*_*v*−1_,*x*_*v*−2_,*x*_*v*−3_ with radii *d*_*v*,*v*−1_,*d*_*v*,*v*−2_,*d*_*v*,*v*−3_: the first two spheres either do not meet or their intersection is in general a circle, and the intersection of the third sphere with this circle is either empty or consists in general of two points [56]. We can now consider the distances defined on pruning edges in *E*_*P*_, linking *v* to its preceding vertices in order to accept or reject these two points. For each accepted point we recursively call BP with *v* replaced by *v*+1, for all *v*<*n*. When *v*=*n* we have a valid realization of the graph: we save it in *X*, and proceed to complete the recursive search. This yields a search tree which is explored depth-first. The recursion starts after placing the initial triangle 1,2,3 (either arbitrarily or by using BP restricted to subspaces), so this tree starts branching at level 4. It can be proved that, at completion, *X* contains all incongruent (modulo translations and rotations) realizations of *G*.

In the case where $E_{P}=\varnothing $, the search tree is a complete binary tree with 2^*n*−3^ nodes at the *n*-th (and last) level: in other words, its depth is *n* and its width is 2^*n*−3^. This is the worst case, since the BP must explore all of the nodes in the tree, and proves that the BP (and hence the *i*BP, since it generalizes the BP) is an exponential-time algorithm in *n*.

When $E_{P}\not =\varnothing $, it was shown that *X* almost always contains a number of solutions which is either zero or a power of two [55]; this discovery led to a set of results where the BP search tree width can be kept polynomial in *n* during the search [53]. Since the exponential behavior is only due to the tree width, this yields a set of cases where the BP is actually fixed-parameter tractable (FPT). Throughout all our experiments with protein data we were always able to fix the parameter controlling the exponential growth of the tree width to a universal constant, which makes BP “polynomial on proteins” (this is an informal statement — the precise statement is given in [53]).

### Intervals and discrete distance sets

The theory supporting the case where *d* might map edges to discrete sets of distance values or intervals, which is the case treated in this paper, is not so clearly understood yet. As it generalizes the simpler case sketched above, in a certain sense it inherits its properties, but this is an oversimplification: for instance, if all intervals are [ 0,*∞*], it is obvious that the problem is easy independently of the graph topology, since every realization is valid.

Some bounds on the cardinality of *X* in the presence of discrete sets and intervals are given in [55]. Our understanding is that if the intervals are small enough, the theory which led to fixed-parameter tractability goes through with few changes, but we have no way so far of establishing an aprioristic maximum width for the intervals. If the intervals are very large the problem might become tractable, as mentioned above, for the purposes of finding at least one solution. The *i*BP would still behave exponentially, however.

## Results and discussion

We applied the presented algorithm to three examples of proteins displaying *α* helical secondary structures. Before presenting the obtained results, we emphasize that the method proposed here has a completely different philosophy than classical optimization approaches commonly used in the field of NMR structure determination. In the present approach, each constraint is treated in the strict sense, that is, no violation, however small, is tolerated. This is why we consistently use the word *constraint* in the paper. This is what potentially allows us to systematically explore the entire search space. However, the use of the procedure demands that the data have been pre-processed accordingly, and all geometric inconsistencies that exist in three–dimensional space have been removed.

For the proteins studied here, if one includes the ensemble of NMR interval distance constraints stored in the.mr file at the Protein Data Bank (PDB) [57] as well as all pruning devices described above, all solutions are pruned out, indicating that no solution to the distance geometry problem exists with the deposited data. This is not really surprising, since the optimization algorithms generally used in NMR structure determination are based on optimization of a target function or hybrid energy rather than on strict constraint satisfaction. That is, there is always a phase where the algorithm tries to find a trade-off when inconsistencies exist between constraints. The optimization thus produces solutions in which chemical and NMR constraints are optimized, but in which small violations are always present. These inconsistencies are present in any structure determination, in particular because distance constraints are imprecise, due to experimental limitations.

Since the data in the PDB for the examples presented here were not pre-processed the way our algorithm requires, we decided to use a subset of the stored data sets: the definition of *α*-helix regions and a few long-range distance constraints arbitrary selected from the set of NMR constraints for structures with more than one *α*-helix. In order to further reduce the risk of all solutions being pruned, we used tolerance values for atomic positions and angles between atoms (Table 2).

**Table 2:** **Analysis of conformations obtained by the branch-and-pruning algorithm on the three proteins targets: 2JMY, 2KXA and 2KSL**

**Proteins**	**2JMY**	**2JMY_1**	**2JMY_2**	**2KXA**	**2KSL**
Number of					
residues	15	15	15	24	51
Number of					
vertices	107	107	107	170	359
Definition					
of *α* helices	1-15	3-13	5-11	1-11, 13-23	4-11, 13-27,
					29-36, 41-50
Position					
tolerance (Å)	0.2	0.2	0.2	0.2	0.2
Angle					
tolerance (°)	2	2	2	4	4
*b* value	4	4	4	8	4
Number of					
long-range					
constraints	0	0	0	1	3
Number of saved					
conformations	1	10000	10000	10000	10000
Number of generated					
conformations	1	633,937	928,399	3,380,964	491,498
CPU time	-	1 min	1 min	25 min	31 min
Number of violated					
constraints (> 1Å)	0	4.0 ± 2.1	11.6 ± 3.6	9.6 ± 2.9	12.8 ± 1.1
Maximum					
violation (Å)	0	3.3 ± 1.4	4.8 ± 0.7	3.7 ± 1.0	8.1 ± 0.6
Mininum RMSD					
from PDB structure (Å)	1.4	1.3	2.1	1.1	3.0
RMSD from PDB structure					
for minimum violated					
conformations (Å)	1.4	2.9	2.8	1.3	3.5
PROCHECK					
core residues (%)	100	65.7 ± 25.9	49.2 ± 7.6	60.4 ± 8.1	76.9 ± 2.4
allowed residues (%)	0	17.9 ± 9.7	40.9 ± 8.3	39.6 ± 8.0	21.3 ± 2.8
gen.allow. residues (%)	0	3.6 ± 4.8	9.9 ± 7.2	0.0 ± 0.0	1.9 ± 1.7
disall. residues (%)	0	0.0 ± 0.0	0.0 ± 0.0	0.0 ± 0.0	0.0 ± 0.0

2JMY_1 and 2JMY_2 correspond to the target 2JMY with shorter definitions of *α* helices. The total number of generated conformations is given, along with the number conformations filtered according to RMSD values.

The three examples we chose to illustrate the algorithm display an increasing structural complexity: (i) a single *α* helix, corresponding to the structure of peptide CM15 determined in micelles (PDB id: 2JMY [58]), (ii) an *α* helical hairpin (PDB id: 2KXA [59]), (iii) the insecticidial toxin TAITX-1a, formed as a bundle of four *α* helices, restrained by three disulphide bridges (PDB id: 2KSL). The main characteristics of the studied proteins are given in Table 2. All three examples were originally determined by NMR spectroscopy, and the corresponding constraint lists are available from the PDB. The analysis by PROCHECK [60] of the Ramachandran diagram of these three PDB structures shows that more than 85% of the residues are located in the core region. For 2KXA and 2KSL, more than 95% of the residues are located in the core and allowed region, whereas in 2JMY, 7% of the residues are located in the generously allowed region. For 2KXA, one PRO residue was replaced by an ALA, as the PRO cycle has not yet been included in the current version of the *i*BP algorithm.

We generated conformations using the branching phase and the pruning devices described above. The long-range constraints added for the calculations of 2KXA and 2KSL, are:

- for 2KXA, one constraint between H *α* hydrogen and carbonyl oxygen of Ala-5 and Met-17, enforcing the pairing of the two *α*-helices,
- for 2KSL, three constraints between Carbons *β* of Cys-7 and Cys-37, of Cys-23 and Cys-33 and of Cys-26 and Cys-46, corresponding to the formation of the three disulphide bridges.

For all calculations, except the one of 2JMY with the *α* helix defined along the whole sequence, the obtained conformations were filtered according to the coordinate root mean-squared deviation (RMSD: 1.5 Å) with respect to the previously obtained conformation in the *i*BP procedure. Enforcing an RMSD value larger than 1.5 Å between two successively stored conformations, avoids an oversampling of the conformational space. Each calculation was stopped after storing 10000 filtered conformations. For our three examples, five calculations were performed in total: three on 2JMY with different definitions of the *α* helix (residues 1-15, 3-13 and 5-11), and one each for 2KXA and 2KSL. For the first calculation on 2JMY, one conformation was obtained and saved. The second and third calculations on 2JMY were quite short, of the order of minutes (Table 2), which is due to the small size of the corresponding tree. For the 2KXA and 2KSL calculations, 10000 conformations were obtained in about 30 mins of calculation. Large total numbers of conformations were generated: this number increases from ∼634,000 (2JMY_1) up to ∼3,400,000 (2KXA) with the size of the considered problem, depending on the number of residues and on the number of constraints. Despite 2KSL being the largest example, the second smallest number of conformations was generated, which is the sign of a severe pruning arising from a rather restricted conformational space.

The reliability of the obtained conformations was checked in three ways. First, the whole set of NMR constraints deposited along with the PDB entries and involving backbone hydrogens, were probed on the conformations. Second, the quality of the obtained conformations was checked using PROCHECK [60] analysis of the Ramachandran plot. Third, the obtained conformations were clustered with an unsupervised clustering method, namely the self-organizing map or SOM [61-64], in order to investigate the properties of sampled conformations.

The agreement of the obtained conformations with the backbone NMR constraints deposited with the PDB structures was checked by calculating the distances between the backbone hydrogens in each obtained conformation. The distances larger than the upper bound of the constraint correspond to violations of this constraint. The mean number of violated constraints along with the mean value of the difference to the upper bound for these constraints were calculated on all conformations (Table 2). For the 2JMY calculation with the 1-15 *α* helix definition, no violation of the NMR constraints could be observed. As expected, when the *α* helix definition is reduced (2JMY_1 and 2JMY_2), the average number of violations increases as well as the average maximum violation. Not surprisingly, the most violated constraints involve residues located at the N and C terminal parts of the *α*-helix, TRP-2, PHE-5, LYS-3, LYS-6 and VAL-11, VAL-14, LEU-15 for 2JMY_1 and 2JMY_2. The largest violations and number of violations are of the same order or value for 2KXA than for 2JMY_1 and 2JMY_2. In contrast, the largest violations and number of violations are observed for 2KSL and involve residues CYS-33, GLU-34, PHE-38, TYR-43. Such over-restraining of NMR structures have been put in evidence in the past, through molecular dynamics simulations [65] and analysis of the structure quality [66].

The average number of violations is similar for 2JMY_2, 2KXA and 2KSL, but the average maximum violation for 2KSL is twice as large as that for 2JMY_2 and 2KXA. This might be due to the very restrained conformations of 2KSL, which contain three disulphide bridges. Due to this restrained conformation, the NMR constraint list is probably more prone to contain inconsistencies, and large mechanical strain can be stored in the structure if one uses an optimization procedure such as simulated annealing. In contrast, no mechanical strain whatsoever is generated by the *i*BP algorithm, and the obtained conformations might have a stronger tendency to deviate from the PDB conformations.

For each example, the obtained conformations were compared to the first conformation deposited in the PDB. Minimum RMSD values in the range 1.1-2.1 Å were obtained for all targets, except 2KSL for which the minimum RMSD value was 3.0 Å. Thus the Branch-and-Prune algorithm was able to capture conformations close to the PDB conformations, the larger value obtained for 2KSL arising from the larger mechanical strain quoted above.

For each calculation, the conformation displaying the smallest number of NMR constraint violations was compared to the first conformation deposited in the PDB. The RMSD values are smaller than 1.5 Å for 2JMY and 2KXA. This shows that, in the context of the *i*BP algorithm, the measured NMR constraints also push the structure toward the PDB structure. For 2JMY_1 and 2JMY_2, the RMSD value increases since the definition of the *α* helical region is shorter. For 2KSL, the conformation displaying the smallest number of constraint violations, displays an RMSD of 3.5Å with the PDB first conformation, which agrees with the maximum number of violations observed for this protein and with the minimum RMSD with the PDB structure analyzed above.

From the PROCHECK [60] analysis, the percentage of residues located in core and allowed Ramachandran regions, is larger than 95% for all targets except 2JMY_1, 2JMY_2, for which the percentages are about 80% due to the reduced definition of the *α* helix. For all targets, the percentage of residues in disallowed regions is equal to zero. The relatively important percentage of residues located in the allowed region may arise from the systematic exploration performed by the Branch-and-Prune algorithm, the strict nature of the constraints, and the nature of the pruning devices.

In order to further probe the robustness of the proposed algorithm, *i*BP calculations on 2KXA and 2KSL have been performed, using input data degraded in the following way: (i) the length of each *α* helix has been reduced by 1 residues at each extremity, (ii) the lower and upper bounds of the long-range distance constraints have been increased by 0.5 Å. The introduction of this noise into the *α* helical and long-range constraints makes the *i*BP solution moving apart from the PDB structure, as the minimum RMSD to PDB structure changes from 1.1 to 2 Å for 2KXA, and from 3.0 to 4.3 Å for 2KSL. Nevertheless, the quality of the Ramachandran diagram remains satisfying, with 93.3% and 95.4% of the residues located in the core and allowed regions of the Ramachandran plot for 2KXA and 2KSL.

**Figure 6:**
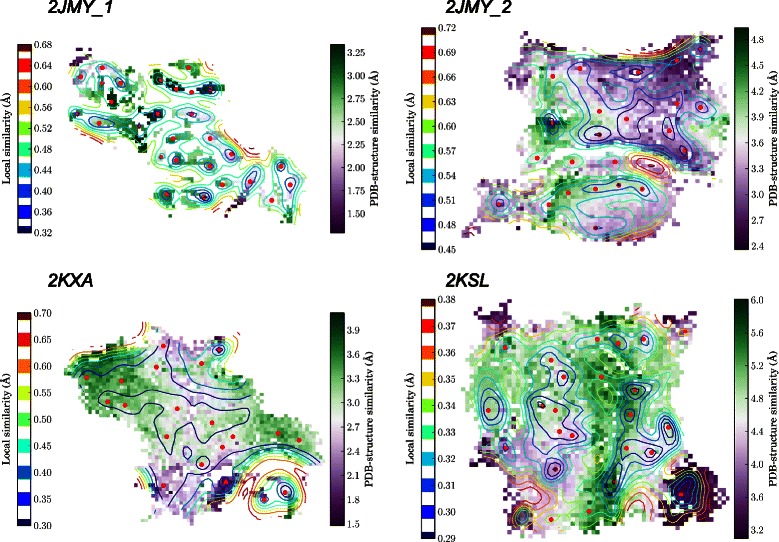
**Clustering of the conformations obtained by the** ***i*** **BP algorithm.** Self-organizing maps describing the clustering of the conformations obtained by the *i*BP algorithm on 2JMY, 2KXA and 2KSL. The contour plots (lines) represent the local similarity between the clustered conformations. The color scales (on plot left) extend from blue to red (from very similar to very dissimilar conformations). The small red points are drawn on the SOM neuron for which the largest local similarity is observed between conformations. Each SOM neuron is colored according to the average value of the coordinates RMSD of the neuron conformations with respect to the PDB structure. The color scales extend (on plot right) from purple to green (from very similar to very dissimilar to the PDB structure). The similarity between SOM neurons as well as the RMSD to the PDB structure are expressed in Å for comparison purposes.

The conformations were clustered using a self-organizing map (SOM) approach [62,63], on which the coordinate RMSD values between the conformers obtained by Branch-and-Prune and the corresponding PDB structure, were projected on the SOMs (Figure 6). These RMSD values lay in the 1.3-3.2 Å range for 2JMY_1, in the 2.4-4.9 Å range for 2JMY_2, in the 1.5-4.0 Å range for 2KXA, and in the 3.2-6.0 Å for 2KSL.

In the SOMs for the four calculations (Figure 6), the RMSD values are colored according to their RMSD from the PDB entry, violet color indicating values smaller than the median value of the sampled RMSD value, green color indicating RMSD values larger than this median value. For 2JMY_1, 2KXA and 2KSL, a larger number of neurons of the SOMs belongs to the second group, which is the sign of an enhanced sampling of the conformational space with respect to the region sampled by simulated annealing. For 2JMY_2, the inverse picture is observed, which may arise from the more limited conformational space available to be sampled for a unique *α*-helix.

**Figure 7:**
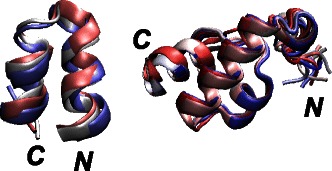
**Superimposed 2KXA and 2KSL conformations.** Superimposition of 2KXA and 2KSL conformations extracted from the SOM, as the ones displaying the minimum coordinates RMSD with respect to the first conformer of the corresponding PDB structures. The N and C terminal extremities are labeled, and the conformations, drawn in cartoon, are colored from blue to red, according to the conformational index.

In 2KSL and 2KXA SOMs, the protein conformations corresponding to the region displaying the smallest coordinate RMSD values with respect to the PDB structure, were extracted (Figure 7). These sets of conformers are similar to the superimposed conformations obtained in a usual NMR calculation.

## Conclusions

We proposed here a Branch-and-Prune algorithm (*i*BP) to solve the Distance Geometry Problem, in order to sample exhaustively the conformational space of the backbone of *α*-helical proteins. The *i*BP algorithm bears a very slight reminiscence to variable target function approaches for example implemented in DISMAN [67], due to the sequential nature of introducing constraints and non-bonded interactions. However, the precise way of introducing the constraints and non-bonded interactions differs significantly, and DISMAN does not systematically search space but is an optimization approach.

We introduced new pruning devices integrated in the *i*BP algorithm for DGP with intervals and we tested our *i*BP implementation on the backbones of *α*-helical proteins. Several pruning devices have been designed to enforce amino-acid chirality, *α*-helix geometry and van der Waals steric hindrance. The algorithm allowed to efficiently reconstruct backbone conformations of three *α*-helical peptides, of various sizes, and for which the structure were previously solved by NMR. The obtained solutions satisfy most of the NMR constraints involving backbone hydrogen bonds, and display very acceptable Ramachandran statistics. The present work represents a first successful step on the way to reconstruct protein structures using a branch-and-prune algorithm applied to the Distance Geometry problem.

Applications where this approach could have significant advantages are cases where there are few distances defining the tertiary structure of a protein, where it is important to characterize the space of all solutions. It might also be useful as part iterative automated assignment algorithms such as ARIA [68], CYANA [69] or UNIO [70], where in a first iteration all solutions compatible with a few unambiguous long-range constraints could be generated to reduce the ambiguity of the remaining constraints. Another application of the approach proposed here would be to provide input molecular conformations to model the structure of multi-subunit complexes into an electron microscopy density map [71].

Some limitations of the current version of *i*BP prevent for the moment its use with real nuclear Overhauser effect (NOE) data. These limitations are the use of unambiguous distance constraints, the non-inclusion of protein side-chains, the loss of information intervals and the appropriate weighting of the various constraints in order to overcome the inconsistencies contained among the whole constraint set. Protein side-chains can be added to the protein backbone afterward. The discretization of circle arcs could be tackled using algebraic geometry and geometric algebra approaches [72]. The Bayesian approach [73] developed for the objective weighting of various NMR contraints according to the data quality could be used to alleviate the inconsistency problems. The use of unambiguous distance constraints is probably the most unavoidable aspect of the current set-up of the algorithm.
